# Hydro­thermal synthesis and crystal structure of a new lanthanum(III) coordination polymer with fumaric acid

**DOI:** 10.1107/S2056989015007008

**Published:** 2015-04-22

**Authors:** Hayet Anana, Chahrazed Trifa, Sofiane Bouacida, Chaouki Boudaren, Hocine Merazig

**Affiliations:** a Unité de Recherche de Chimie de l’Environnement et Moléculaire Structurale, CHEMS, Université Constantine 1, 25000 , Algeria; b Département Sciences de la Matière, Faculté des Sciences Exactes et Sciences de la Nature et de la Vie, Université Larbi Ben M’hidi, Oum El Bouaghi, Algeria

**Keywords:** crystal structure, hydro­thermal synthesis, lanthanum(III) coordination polymer, fumaric acid

## Abstract

The title compound, poly[di­aqua­tris­(μ_4_-but-2-enedioato)(μ_2_-but-2-enedioic acid)dilanthanum(III)], [La_2_(C_4_H_2_O_4_)_3_(C_4_H_4_O_4_)(H_2_O)_2_]_*n*_, was synthesized by the reaction of lanthanum chloride penta­hydrate with fumaric acid under hydro­thermal conditions. The asymmetric unit comprises an La^III^ cation, one and a half fumarate dianions (*L* ^2−^), one a half-mol­ecule of fumaric acid (H_2_ *L*) and one coordinated water mol­ecule. Each La^III^ cation has the same nine-coordinate environment and is surrounded by eight O atoms from seven distinct fumarate moieties, including one proton­ated fumarate unit and one water mol­ecule in a distorted tricapped trigonal–prismatic environment. The LaO_8_(H_2_O) polyhedra centres are edge-shared through three carboxyl­ate bridges of the fumarate ligand, forming chains in three dimensions to construct the MOF. The crystal structure is stabilized by O—H⋯O hydrogen-bond inter­actions between the coordin­ated water mol­ecule and the carboxyl­ate O atoms, and also between oxygen atoms of fumaric acid

## Related literature   

For general background to metal coordination polymers, see: Fujita *et al.* (1994 ▸); Bénard *et al.* (2000 ▸); Zhang *et al.* (2000 ▸). For structures involving fumarate ligands and transition metals, see: Dalai *et al.* (2002 ▸); Xie *et al.* (2003 ▸); Devereux *et al.* (2000 ▸). For rare earth fumarates, see: Zhang *et al.* (2006 ▸); Li & Zou (2006 ▸); Liu *et al.* (2011 ▸). For reported La—O distances, see: Dan *et al.* (2005 ▸).
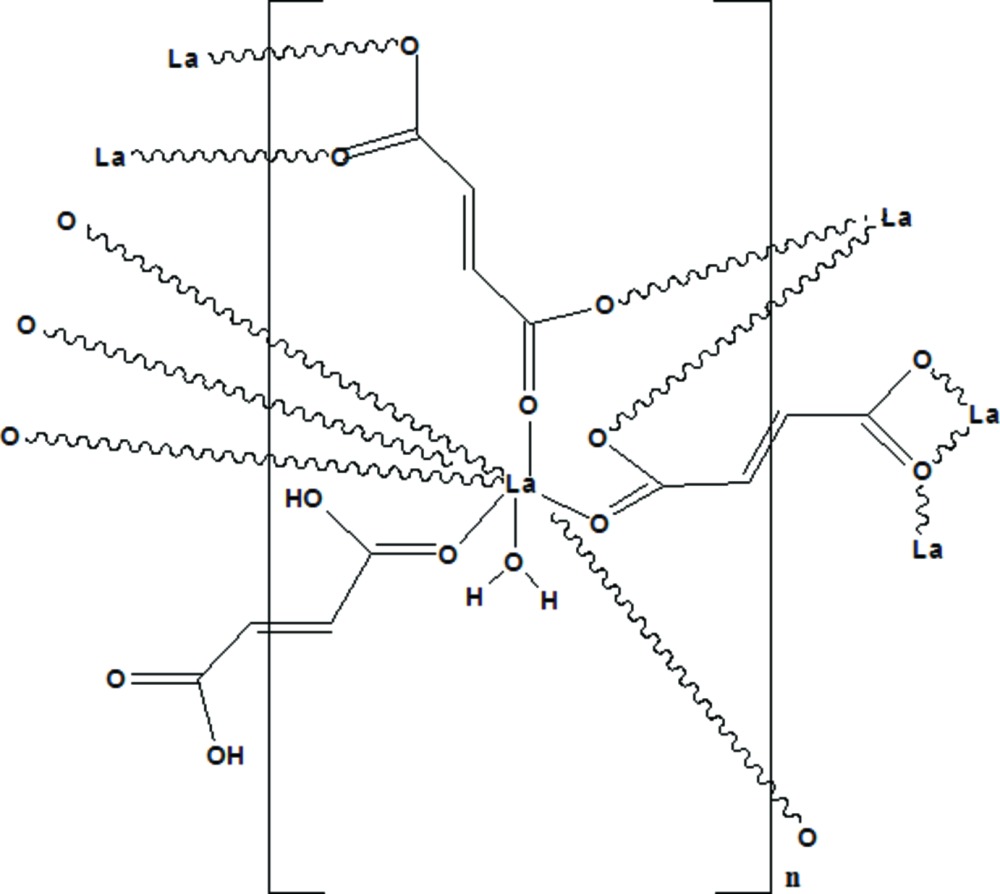


## Experimental   

### Crystal data  

[La_2_(C_4_H_2_O_4_)_3_(C_4_H_4_O_4_)(H_2_O)_2_]
*M* *_r_* = 386.05
Monoclinic, 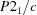

*a* = 8.4299 (5) Å
*b* = 14.6789 (8) Å
*c* = 8.8096 (5) Å
β = 103.318 (3)°
*V* = 1060.80 (11) Å^3^
*Z* = 4
Mo *K*α radiation
μ = 4.07 mm^−1^
*T* = 295 K
0.12 × 0.11 × 0.08 mm

### Data collection  

Bruker APEXII diffractometer
Absorption correction: multi-scan (*SADABS*; Sheldrick, 2002 ▸) *T*_min_ = 0.677, *T*_max_ = 0.796
17677 measured reflections
4523 independent reflections
3901 reflections with *I* > 2σ(*I*)
*R*_int_ = 0.027

### Refinement  

*R*[*F* ^2^ > 2σ(*F* ^2^)] = 0.020
*wR*(*F* ^2^) = 0.043
*S* = 1.02
4523 reflections
171 parameters
H atoms treated by a mixture of independent and constrained refinement
Δρ_max_ = 2.06 e Å^−3^
Δρ_min_ = −0.67 e Å^−3^

### 

Data collection: *APEX2* (Bruker, 2011 ▸); cell refinement: *SAINT* (Bruker, 2011 ▸); data reduction: *SAINT*; program(s) used to solve structure: *SIR2002* (Burla *et al.*, 2005 ▸); program(s) used to refine structure: *SHELXL97* (Sheldrick, 2008 ▸); molecular graphics: *ORTEP-3 for Windows* (Farrugia, 2012 ▸) and *DIAMOND* (Brandenburg & Berndt, 2001 ▸); software used to prepare material for publication: *WinGX* (Farrugia, 2012 ▸) and *CRYSCAL* (T. Roisnel, local program).

## Supplementary Material

Crystal structure: contains datablock(s) I. DOI: 10.1107/S2056989015007008/lh5759sup1.cif

Structure factors: contains datablock(s) I. DOI: 10.1107/S2056989015007008/lh5759Isup2.hkl

**Fig. 1.**
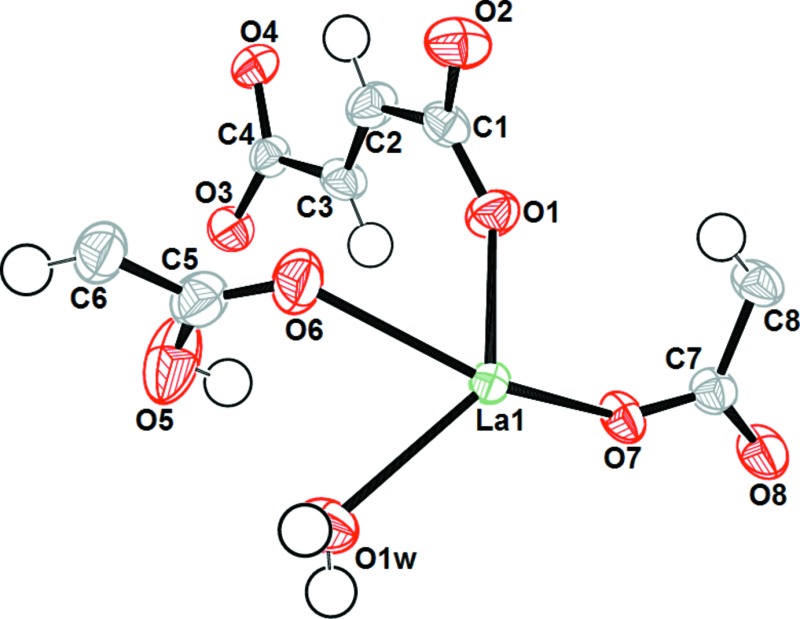
An ORTEP-3 (Farrugia, 2012) drawing of (I), with the atom-numbering scheme. Displacement ellipsoids are drawn at the 50% probability level.

ORTEP-3 . DOI: 10.1107/S2056989015007008/lh5759fig1.tif
An *ORTEP-3* (Farrugia, 2012) drawing of (I), with the atom-numbering scheme. Displacement ellipsoids are drawn at the 50% probability level.

**Fig. 2.**
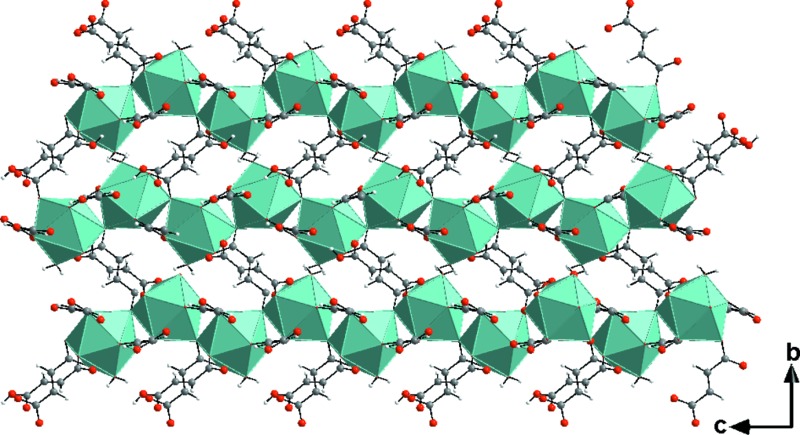
A packing diagram of (I), showing the two-dimensional layered framework structure.

. DOI: 10.1107/S2056989015007008/lh5759fig2.tif
A packing diagram of (I), showing the two-dimensional layered framework structure.

**Fig. 3.**
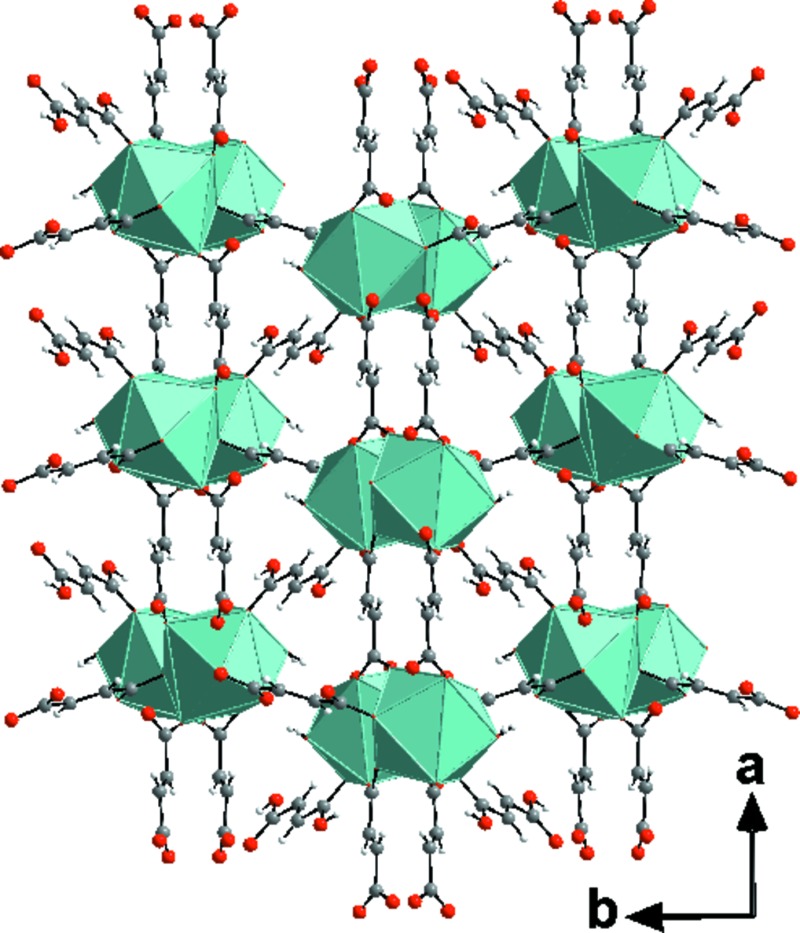
A packing diagram of (I), showing the three-dimensional open-framework structure.

. DOI: 10.1107/S2056989015007008/lh5759fig3.tif
A packing diagram of (I), showing the three-dimensional open-framework structure.

CCDC reference: 1058359

Additional supporting information:  crystallographic information; 3D view; checkCIF report

## Figures and Tables

**Table 1:** Hydrogen-bond geometry (, )

*D*H*A*	*D*H	H*A*	*D* *A*	*D*H*A*
O1*W*H1*W*O8^i^	0.80(3)	2.06(3)	2.7995(19)	154(3)
O1*W*H2*W*O4^ii^	0.75(3)	2.17(3)	2.8913(18)	163(3)
O5H5O2^iii^	0.82	1.85	2.655(2)	167

Symmetry codes: (i) 
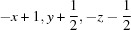
; (ii) 
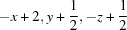
; (iii) 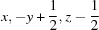.

## supplementary crystallographic information

### S1. Comment

Coordination polymers of metal cations with organic multifunctional ligands have been received increasing interest, for these coordination polymers have one-, two-, three dimensional structures as well as potential applications as catalysts, magnetic and porous materials (Fujita *et al.*, 1994; Bénard *et al.*, 2000; Zhang *et al.*, 2000). Multi-carboxyle ligands are useful to construct unique architectures of metal-coordination polymers. The synthesis of novel lanthanide polymers and studies on luminescent, electric and magnetic properties of the compounds are of interest. Some metal coordination polymers using fumaric acid as ligand have been reported in the literature containing transition metals Cu (Dalai *et al.*, 2002), Zn (Xie *et al.*, 2003), and Mn (Devereux *et al.*, 2000). A series of rare earth fumarate complexes have also been reported (Zhang *et al.*, 2006; Li & Zou., 2006; Liu *et al.*, 2011). Hydrothermal synthesis has some advantages over conventional methods for the formation of a polymer framework with higher dimensions. Fumaric acid was used to synthesize a new lanthanum (III) coordination polymer, [La_2_(C_4_H_2_O_4_)_3_(C_4_H_4_O_4_)(H_2_O)_2_]n, (I), by using hydrothermal synthesis method and the crystal structure is reported in the present article.

The structure of the asymmetric unit of the title complex is shown in Fig. 1. It comprises a La^III^ cation, 1.5 fumarate dianions (*L*^2-^), 0.5 fumaric acid (H_2_*L*) and one water ligand. Overall there are three types of La—O bridging modes in (I), the fumarate dianion exhibits full monodentate and µ2-oxo-bridged chelating patterns, respectively, whereas the fumaric acid shows a double monodentate coordination mode. The La^III^ cation is sited within a distorted tricapped trigonal prism defined by nine O atoms derived from seven different bridging ligands and a coordinated water molecule. One of the carboxylate groups, derived from *L*^2-^, is chelating, and the remaining six carboxylates coordinate in a monodentate mode. The average La—O bond distance of LaO_8_(H_2_O) polyhedra is 2.56 Å; the shortest La—O separation is 2.4510 (12) Å, resulting from the La1—O1 bond of a bridging carboxylate, and the longest is 2.7696 (12) Å for La1—O7 from the edge-sharing La—O bond. Other distances of La—O(fum) vary in the range of 2.4963 (12)–2.6117 (13) Å, comparable to the usual La—*O*(carboxylate) bonds reported (Dan *et al.*, 2005). The LaO_8_(H_2_O) coordination polyhedra are edge-shared through one monodentate carboxylate O atoms (O7) and two bidentate carboxylate groups (O3—C4—O4 and O1—C1—O2) to generate infinite lanthanum-oxygen chains (Fig. 2). The adjacent lanthanum (III) centres have a general separation of 4.739 Å. Furthermore, the one-dimensional infinite chains are linked together with monodentate fumarate ligands to form a two-dimensional layered paralell to the crystallographic (100) (Fig.2), and the shortest interlayer distance of La···La is 8.430 Å (calculated between the two lanthanum atom centres). This type of organic-inorganic layered structure has been reported of the lanthanide fumarates: [Ln_2_(fum)_3_(H_2_fum)(H_2_O)_2_ (Ln: Ce or Nd)] (Zhang *et al.*, 2006). Finally, the two-dimensional layered structure is further constructed into a three-dimensional open framework by the ligands (Fig.3). The crystal is stabilized by hydrogen bond interactions between the coordinated water and carboxylate O atoms.

### S2. Experimental

All chemicals were purchased from commercial sources and used as received without further purification. The title compound, was synthesized by using a hydrothermal method. Typically mixtures of fumaric acid (1 mmol, 0.116 g), lanthanum (III) chloride pentahydrate (0.5 mmol, 0.185 g) were suspended in H_2_O (*ca* 10 ml). The mixture was then placed in a Teflon lined autoclave, sealed and heated to 413 K for 2 days. The reactor was cooled to room temperature over a period of 1 h. The light brown crystals suitable for X-ray diffraction were filtered, washed with water and dried in air.

### S3. Refinement

All non-H atoms were refined with anisotropic atomic displacement parameters. The remaining H atoms were located in difference Fourier maps but introduced in calculated positions and treated as riding on their parent atom (C and O atoms) with C—H = 0.93 Å and O—H = 0.82 Å with *U*_iso_(H) = 1.2 or 1.5*U*_eq_(C,O). H atoms of the water molecule were located in difference Fourier maps and refined isotropically.

### Crystal data

**Table:**

[La_2_(C_4_H_2_O_4_)_3_(C_4_H_4_O_4_)(H_2_O)_2_]	*F*(000) = 736
*M**_r_* = 386.05	*D*_x_ = 2.417 Mg m^−^^3^
Monoclinic, *P*2_1_/*c*	Mo *K*α radiation, λ = 0.71073 Å
Hall symbol: -P 2ybc	Cell parameters from 7844 reflections
*a* = 8.4299 (5) Å	θ = 2.8–34.5°
*b* = 14.6789 (8) Å	µ = 4.07 mm^−^^1^
*c* = 8.8096 (5) Å	*T* = 295 K
β = 103.318 (3)°	Prism, brown
*V* = 1060.80 (11) Å^3^	0.12 × 0.11 × 0.08 mm
*Z* = 4	

### Data collection

**Table:**

Bruker APEXII diffractometer	3901 reflections with *I* > 2σ(*I*)
Graphite monochromator	*R*_int_ = 0.027
CCD rotation images, thin slices scans	θ_max_ = 34.6°, θ_min_ = 2.5°
Absorption correction: multi-scan (*SADABS*; Sheldrick, 2002)	*h* = −13→13
*T*_min_ = 0.677, *T*_max_ = 0.796	*k* = −23→22
17677 measured reflections	*l* = −14→14
4523 independent reflections	

### Refinement

**Table:**

Refinement on *F*^2^	Primary atom site location: structure-invariant direct methods
Least-squares matrix: full	Secondary atom site location: difference Fourier map
*R*[*F*^2^ > 2σ(*F*^2^)] = 0.020	Hydrogen site location: inferred from neighbouring sites
*wR*(*F*^2^) = 0.043	H atoms treated by a mixture of independent and constrained refinement
*S* = 1.02	*w* = 1/[σ^2^(*F*_o_^2^) + (0.0198*P*)^2^ + 0.4033*P*] where *P* = (*F*_o_^2^ + 2*F*_c_^2^)/3
4523 reflections	(Δ/σ)_max_ = 0.003
171 parameters	Δρ_max_ = 2.06 e Å^−^^3^
0 restraints	Δρ_min_ = −0.67 e Å^−^^3^

### Special details

**Table:**

Geometry. All e.s.d.'s (except the e.s.d. in the dihedral angle between two l.s. planes) are estimated using the full covariance matrix. The cell e.s.d.'s are taken into account individually in the estimation of e.s.d.'s in distances, angles and torsion angles; correlations between e.s.d.'s in cell parameters are only used when they are defined by crystal symmetry. An approximate (isotropic) treatment of cell e.s.d.'s is used for estimating e.s.d.'s involving l.s. planes.
Refinement. Refinement of *F*^2^ against ALL reflections. The weighted *R*-factor *wR* and goodness of fit *S* are based on *F*^2^, conventional *R*-factors *R* are based on *F*, with *F* set to zero for negative *F*^2^. The threshold expression of *F*^2^ > σ(*F*^2^) is used only for calculating *R*-factors(gt) *etc*. and is not relevant to the choice of reflections for refinement. *R*-factors based on *F*^2^ are statistically about twice as large as those based on *F*, and *R*- factors based on ALL data will be even larger.

### Fractional atomic coordinates and isotropic or equivalent isotropic displacement parameters (Å^2^)

**Table:**

	*x*	*y*	*z*	*U*_iso_*/*U*_eq_	
C1	0.91794 (19)	0.17630 (11)	0.3525 (2)	0.0094 (3)	
C2	1.09547 (19)	0.16975 (12)	0.3538 (2)	0.0116 (3)	
H2	1.1677	0.1528	0.4461	0.014*	
C3	1.1551 (2)	0.18705 (11)	0.22925 (19)	0.0104 (3)	
H3	1.0842	0.2006	0.1344	0.012*	
C4	1.33672 (18)	0.18480 (11)	0.24064 (19)	0.0091 (3)	
C5	0.9759 (2)	0.43916 (12)	0.3056 (2)	0.0136 (3)	
C6	1.0339 (2)	0.50146 (12)	0.4385 (2)	0.0137 (3)	
H6	1.1173	0.5426	0.4366	0.016*	
C7	0.53342 (19)	0.10280 (11)	−0.12552 (19)	0.0100 (3)	
C8	0.5242 (2)	0.04320 (11)	0.0094 (2)	0.0115 (3)	
H8	0.5533	0.0672	0.1098	0.014*	
O1	0.81442 (14)	0.18896 (8)	0.22694 (15)	0.0121 (2)	
O2	0.88376 (15)	0.16822 (9)	0.48504 (15)	0.0135 (2)	
O1W	0.68340 (16)	0.47790 (9)	0.02557 (17)	0.0140 (2)	
O3	1.38637 (14)	0.22346 (9)	0.13278 (15)	0.0128 (2)	
O4	1.42800 (14)	0.14400 (8)	0.35629 (14)	0.0113 (2)	
O5	1.05314 (17)	0.44700 (11)	0.19286 (17)	0.0232 (3)	
H5	1.0152	0.4107	0.1232	0.035*	
O6	0.86412 (16)	0.38537 (9)	0.30246 (15)	0.0165 (3)	
O7	0.59366 (15)	0.18252 (8)	−0.09912 (15)	0.0106 (2)	
O8	0.48750 (15)	0.07304 (9)	−0.26313 (14)	0.0145 (2)	
La1	0.631512 (10)	0.309568 (6)	0.097101 (10)	0.00675 (3)	
H1W	0.656 (3)	0.4969 (19)	−0.062 (4)	0.030 (7)*	
H2W	0.651 (3)	0.513 (2)	0.071 (4)	0.036 (8)*	

### Atomic displacement parameters (Å^2^)

**Table:**

	*U*^11^	*U*^22^	*U*^33^	*U*^12^	*U*^13^	*U*^23^
C1	0.0093 (6)	0.0092 (7)	0.0107 (7)	0.0008 (5)	0.0044 (5)	0.0017 (5)
C2	0.0082 (6)	0.0166 (8)	0.0099 (7)	0.0000 (5)	0.0019 (5)	0.0009 (6)
C3	0.0115 (6)	0.0117 (7)	0.0090 (6)	−0.0008 (5)	0.0045 (5)	0.0017 (6)
C4	0.0075 (6)	0.0111 (7)	0.0088 (6)	−0.0007 (5)	0.0021 (5)	−0.0002 (5)
C5	0.0131 (7)	0.0166 (8)	0.0108 (7)	−0.0010 (6)	0.0021 (6)	−0.0006 (6)
C6	0.0142 (7)	0.0153 (8)	0.0113 (7)	−0.0037 (6)	0.0021 (6)	−0.0020 (6)
C7	0.0113 (6)	0.0107 (7)	0.0088 (7)	−0.0007 (5)	0.0036 (5)	−0.0001 (5)
C8	0.0170 (7)	0.0099 (7)	0.0085 (7)	−0.0018 (6)	0.0046 (6)	0.0000 (5)
O1	0.0097 (5)	0.0139 (6)	0.0117 (5)	0.0015 (4)	0.0005 (4)	0.0016 (5)
O2	0.0118 (5)	0.0187 (6)	0.0117 (6)	0.0027 (4)	0.0063 (4)	0.0031 (5)
O1W	0.0171 (6)	0.0110 (6)	0.0141 (6)	0.0019 (4)	0.0040 (5)	0.0016 (5)
O3	0.0105 (5)	0.0175 (6)	0.0113 (6)	−0.0030 (4)	0.0043 (4)	0.0025 (5)
O4	0.0095 (5)	0.0133 (6)	0.0102 (5)	0.0009 (4)	0.0006 (4)	0.0002 (4)
O5	0.0213 (7)	0.0347 (8)	0.0161 (6)	−0.0129 (6)	0.0094 (5)	−0.0119 (6)
O6	0.0180 (6)	0.0191 (7)	0.0118 (6)	−0.0075 (5)	0.0024 (5)	−0.0026 (5)
O7	0.0138 (5)	0.0082 (5)	0.0108 (5)	−0.0019 (4)	0.0051 (4)	−0.0010 (4)
O8	0.0218 (6)	0.0138 (6)	0.0086 (5)	−0.0061 (5)	0.0051 (5)	−0.0017 (5)
La1	0.00657 (4)	0.00743 (4)	0.00650 (4)	−0.00024 (3)	0.00204 (3)	−0.00044 (3)

### Geometric parameters (Å, º)

**Table:**

C1—O1	1.255 (2)	C8—H8	0.93
C1—O2	1.271 (2)	O1W—H1W	0.80 (3)
C1—C2	1.497 (2)	O1W—H2W	0.75 (3)
C2—C3	1.332 (2)	O3—La1^iv^	2.5032 (11)
C2—H2	0.93	O4—La1^v^	2.4963 (12)
C3—C4	1.512 (2)	O5—H5	0.82
C3—H3	0.93	La1—C7^vi^	3.0398 (15)
C4—O3	1.2583 (19)	O6—La1	2.5926 (13)
C4—O4	1.276 (2)	O7—La1	2.5127 (12)
C5—O6	1.225 (2)	O1—La1	2.4510 (12)
C5—O5	1.312 (2)	O1W—La1	2.6117 (13)
C5—C6	1.477 (2)	O2—La1^vi^	2.5631 (11)
C6—C6^i^	1.338 (3)	O7—La1^ii^	2.7696 (12)
C6—H6	0.93	O8—La1^ii^	2.5784 (12)
C7—O8	1.263 (2)	La1—O4^vii^	2.4963 (12)
C7—O7	1.2755 (19)	La1—O3^viii^	2.5032 (11)
C7—C8	1.492 (2)	La1—O2^ii^	2.5631 (11)
C7—La1^ii^	3.0398 (15)	La1—O8^vi^	2.5784 (12)
C8—C8^iii^	1.331 (3)	La1—O7^vi^	2.7696 (12)
			
O1—C1—O2	124.39 (15)	O1—La1—O7	75.61 (4)
O1—C1—C2	120.47 (14)	O4^vii^—La1—O7	70.40 (4)
O2—C1—C2	115.14 (15)	O3^viii^—La1—O7	74.57 (4)
C3—C2—C1	123.19 (16)	O1—La1—O2^ii^	77.47 (4)
C3—C2—H2	118.4	O4^vii^—La1—O2^ii^	96.12 (4)
C1—C2—H2	118.4	O3^viii^—La1—O2^ii^	153.47 (4)
C2—C3—C4	120.64 (15)	O7—La1—O2^ii^	79.32 (4)
C2—C3—H3	119.7	O1—La1—O8^vi^	125.08 (4)
C4—C3—H3	119.7	O4^vii^—La1—O8^vi^	85.20 (4)
O3—C4—O4	124.78 (14)	O3^viii^—La1—O8^vi^	77.55 (4)
O3—C4—C3	116.62 (14)	O7—La1—O8^vi^	145.63 (4)
O4—C4—C3	118.60 (13)	O2^ii^—La1—O8^vi^	128.50 (4)
O6—C5—O5	123.56 (17)	O1—La1—O6	72.00 (4)
O6—C5—C6	121.97 (15)	O4^vii^—La1—O6	137.48 (4)
O5—C5—C6	114.46 (15)	O3^viii^—La1—O6	129.99 (4)
C6^i^—C6—C5	119.8 (2)	O7—La1—O6	138.97 (4)
C6^i^—C6—H6	120.1	O2^ii^—La1—O6	69.69 (4)
C5—C6—H6	120.1	O8^vi^—La1—O6	75.14 (4)
O8—C7—O7	120.91 (15)	O1—La1—O1W	132.35 (4)
O8—C7—C8	120.09 (15)	O4^vii^—La1—O1W	69.95 (4)
O7—C7—C8	118.95 (15)	O3^viii^—La1—O1W	134.19 (4)
O8—C7—La1^ii^	56.95 (8)	O7—La1—O1W	122.55 (4)
O7—C7—La1^ii^	65.65 (8)	O2^ii^—La1—O1W	65.59 (4)
C8—C7—La1^ii^	164.17 (11)	O8^vi^—La1—O1W	66.82 (4)
C8^iii^—C8—C7	122.0 (2)	O6—La1—O1W	67.70 (4)
C8^iii^—C8—H8	119	O1—La1—O7^vi^	77.26 (4)
C7—C8—H8	119	O4^vii^—La1—O7^vi^	126.88 (4)
C1—O1—La1	138.87 (11)	O3^viii^—La1—O7^vi^	67.52 (4)
C1—O2—La1^vi^	136.53 (11)	O7—La1—O7^vi^	132.16 (3)
La1—O1W—H1W	122 (2)	O2^ii^—La1—O7^vi^	131.09 (4)
La1—O1W—H2W	116 (2)	O8^vi^—La1—O7^vi^	48.61 (4)
H1W—O1W—H2W	102 (3)	O6—La1—O7^vi^	62.92 (4)
C4—O3—La1^iv^	138.62 (11)	O1W—La1—O7^vi^	104.86 (4)
C4—O4—La1^v^	136.10 (11)	O1—La1—C7^vi^	100.89 (4)
C5—O5—H5	109.5	O4^vii^—La1—C7^vi^	107.86 (4)
C5—O6—La1	138.30 (12)	O3^viii^—La1—C7^vi^	74.18 (4)
C7—O7—La1	142.04 (10)	O7—La1—C7^vi^	148.44 (4)
C7—O7—La1^ii^	89.54 (9)	O2^ii^—La1—C7^vi^	131.24 (4)
La1—O7—La1^ii^	127.52 (4)	O8^vi^—La1—C7^vi^	24.23 (4)
C7—O8—La1^ii^	98.82 (10)	O6—La1—C7^vi^	63.86 (4)
O1—La1—O4^vii^	146.01 (4)	O1W—La1—C7^vi^	83.41 (4)
O1—La1—O3^viii^	91.46 (4)	O7^vi^—La1—C7^vi^	24.81 (4)
O4^vii^—La1—O3^viii^	79.57 (4)		
			
O1—C1—C2—C3	8.0 (3)	C1—O1—La1—O6	22.16 (15)
O2—C1—C2—C3	−171.99 (16)	C1—O1—La1—O1W	55.44 (17)
C1—C2—C3—C4	176.25 (15)	C1—O1—La1—O7^vi^	−43.17 (16)
C2—C3—C4—O3	−162.53 (16)	C1—O1—La1—C7^vi^	−35.55 (16)
C2—C3—C4—O4	18.0 (2)	C7—O7—La1—O1	70.34 (18)
O6—C5—C6—C6^i^	1.5 (3)	La1^ii^—O7—La1—O1	−124.25 (6)
O5—C5—C6—C6^i^	−178.9 (2)	C7—O7—La1—O4^vii^	−109.49 (18)
O8—C7—C8—C8^iii^	3.3 (3)	La1^ii^—O7—La1—O4^vii^	55.92 (6)
O7—C7—C8—C8^iii^	−174.2 (2)	C7—O7—La1—O3^viii^	−25.29 (17)
La1^ii^—C7—C8—C8^iii^	−71.3 (5)	La1^ii^—O7—La1—O3^viii^	140.12 (7)
O2—C1—O1—La1	70.6 (2)	C7—O7—La1—O2^ii^	150.00 (18)
C2—C1—O1—La1	−109.40 (17)	La1^ii^—O7—La1—O2^ii^	−44.59 (6)
O1—C1—O2—La1^vi^	−9.9 (3)	C7—O7—La1—O8^vi^	−62.2 (2)
C2—C1—O2—La1^vi^	170.08 (11)	La1^ii^—O7—La1—O8^vi^	103.23 (7)
O4—C4—O3—La1^iv^	−33.2 (3)	C7—O7—La1—O6	109.01 (17)
C3—C4—O3—La1^iv^	147.30 (13)	La1^ii^—O7—La1—O6	−85.58 (8)
O3—C4—O4—La1^v^	72.1 (2)	C7—O7—La1—O1W	−158.26 (17)
C3—C4—O4—La1^v^	−108.47 (15)	La1^ii^—O7—La1—O1W	7.15 (8)
O5—C5—O6—La1	−30.8 (3)	C7—O7—La1—O7^vi^	13.0 (2)
C6—C5—O6—La1	148.76 (14)	La1^ii^—O7—La1—O7^vi^	178.394 (15)
O8—C7—O7—La1	154.08 (13)	C7—O7—La1—C7^vi^	−17.1 (2)
C8—C7—O7—La1	−28.5 (3)	La1^ii^—O7—La1—C7^vi^	148.33 (6)
La1^ii^—C7—O7—La1	168.47 (17)	C5—O6—La1—O1	117.06 (19)
O8—C7—O7—La1^ii^	−14.40 (15)	C5—O6—La1—O4^vii^	−42.3 (2)
C8—C7—O7—La1^ii^	163.03 (13)	C5—O6—La1—O3^viii^	−166.68 (17)
O7—C7—O8—La1^ii^	15.68 (17)	C5—O6—La1—O7	77.5 (2)
C8—C7—O8—La1^ii^	−161.72 (12)	C5—O6—La1—O2^ii^	34.12 (18)
C1—O1—La1—O4^vii^	176.91 (14)	C5—O6—La1—O8^vi^	−107.59 (19)
C1—O1—La1—O3^viii^	−109.72 (16)	C5—O6—La1—O1W	−36.94 (18)
C1—O1—La1—O7	176.62 (16)	C5—O6—La1—O7^vi^	−158.3 (2)
C1—O1—La1—O2^ii^	94.61 (16)	C5—O6—La1—C7^vi^	−130.57 (19)
C1—O1—La1—O8^vi^	−33.94 (17)		

Symmetry codes: (i) −*x*+2, −*y*+1, −*z*+1; (ii) *x*, −*y*+1/2, *z*−1/2; (iii) −*x*+1, −*y*, −*z*; (iv) *x*+1, *y*, *z*; (v) *x*+1, −*y*+1/2, *z*+1/2; (vi) *x*, −*y*+1/2, *z*+1/2; (vii) *x*−1, −*y*+1/2, *z*−1/2; (viii) *x*−1, *y*, *z*.

### Hydrogen-bond geometry (Å, º)

**Table:**

*D*—H···*A*	*D*—H	H···*A*	*D*···*A*	*D*—H···*A*
O1*W*—H1*W*···O8^ix^	0.80 (3)	2.06 (3)	2.7995 (19)	154 (3)
O1*W*—H2*W*···O4^x^	0.75 (3)	2.17 (3)	2.8913 (18)	163 (3)
O5—H5···O2^ii^	0.82	1.85	2.655 (2)	167

Symmetry codes: (ii) *x*, −*y*+1/2, *z*−1/2; (ix) −*x*+1, *y*+1/2, −*z*−1/2; (x) −*x*+2, *y*+1/2, −*z*+1/2.
