# Supplementary figures and images for: Hydro­thermal synthesis and crystal structure of a new lanthanum(III) coordination polymer with fumaric acid
Source: Acta Crystallogr E Crystallogr Commun. 2015 Apr 22;71(Pt 5):m114–5. doi: 10.1107/S2056989015007008 (PMC4420125; doi:10.1107/S2056989015007008)

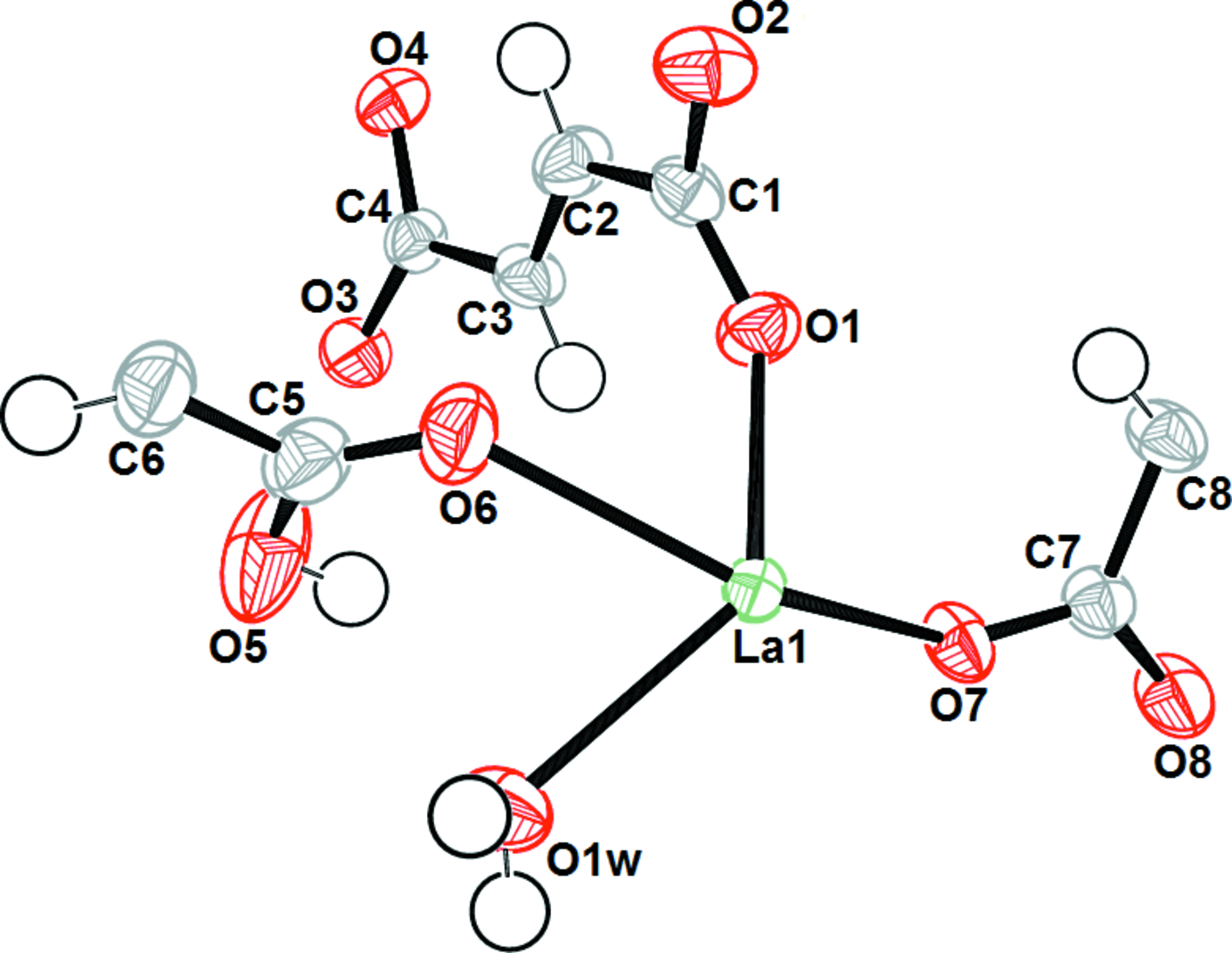

Supplement: Supplementary file 3 [file e-71-0m114-fig1.tif]

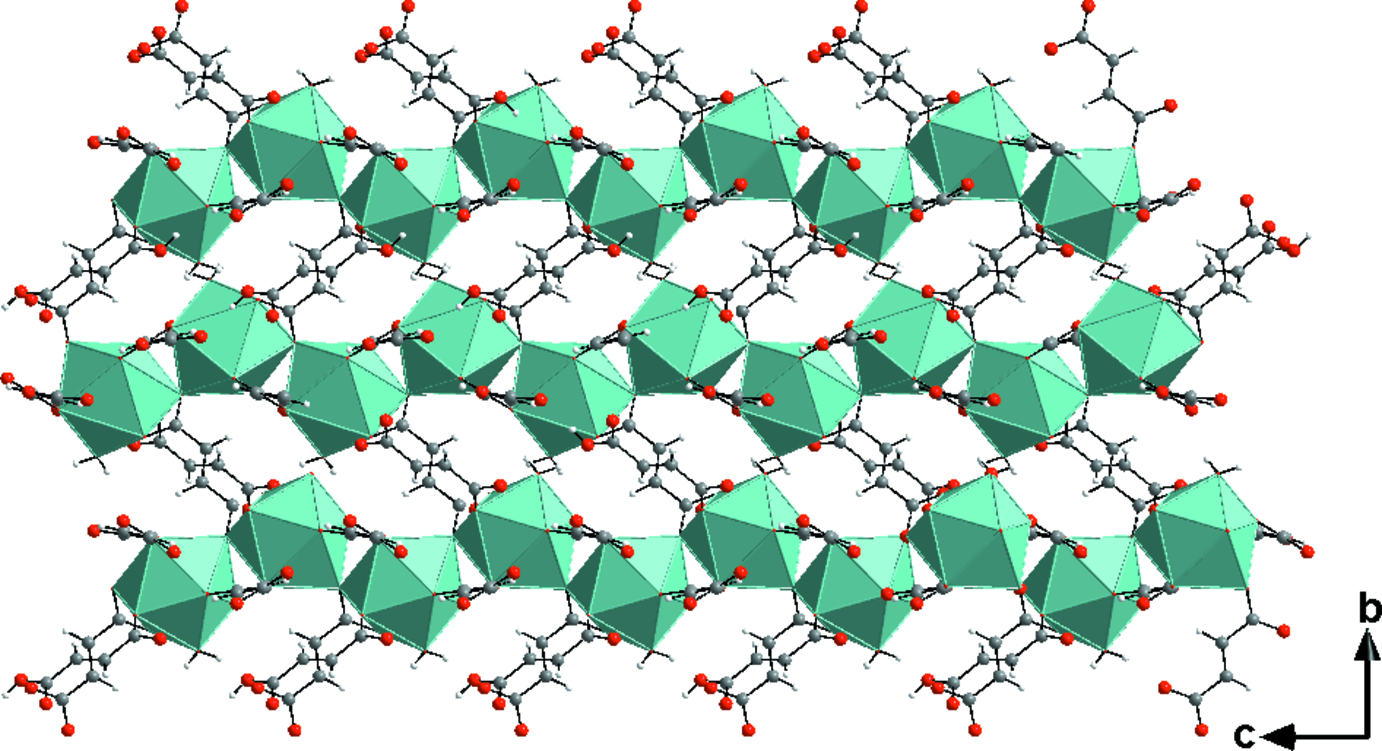

Supplement: Supplementary file 4 [file e-71-0m114-fig2.tif]

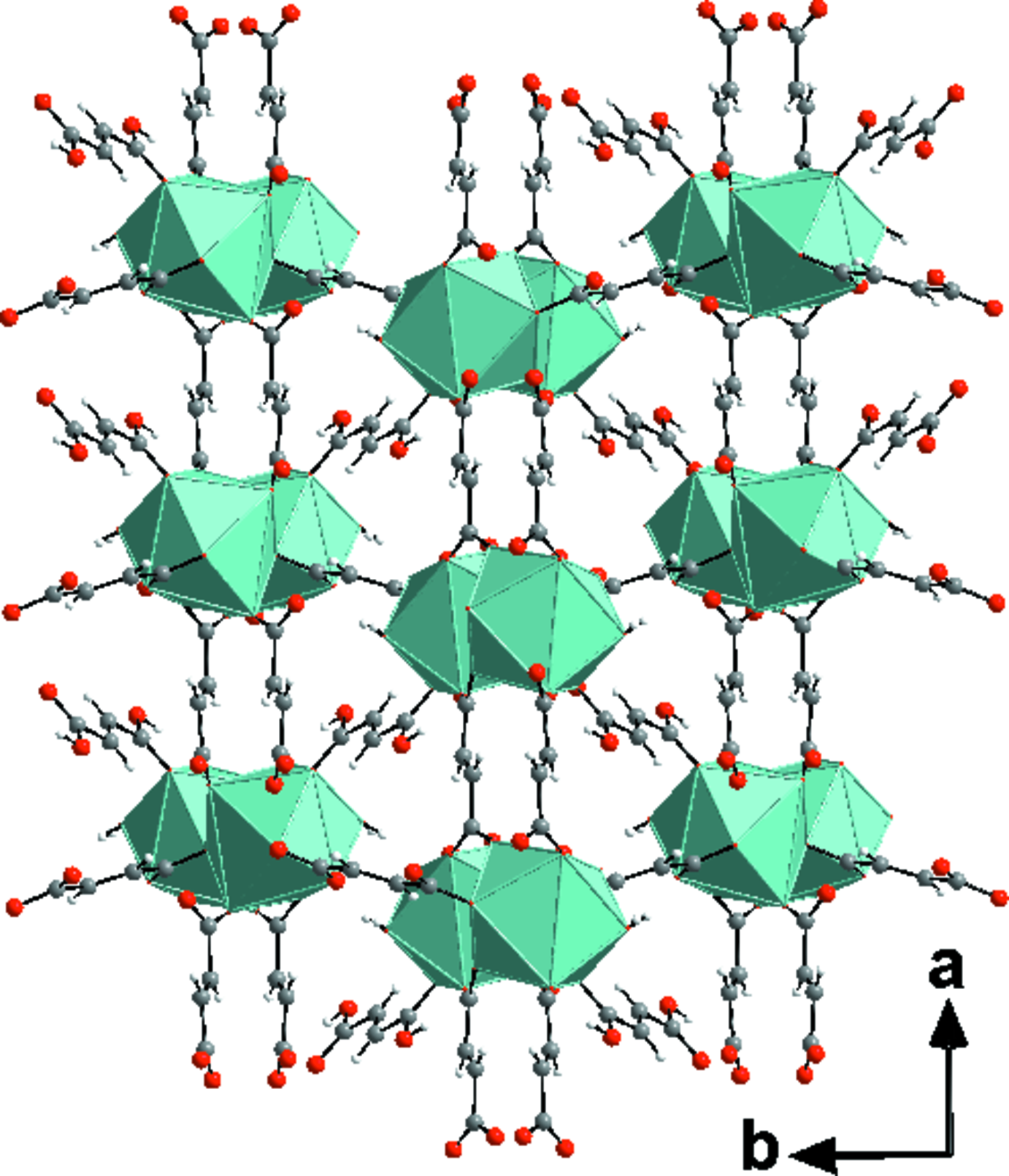

Supplement: Supplementary file 5 [file e-71-0m114-fig3.tif]
